# Enhanced Furfural Production in Deep Eutectic Solvents Comprising Alkali Metal Halides as Additives

**DOI:** 10.3390/molecules26237374

**Published:** 2021-12-04

**Authors:** Eduarda S. Morais, Mara G. Freire, Carmen S. R. Freire, Armando J. D. Silvestre

**Affiliations:** Chemistry Department, CICECO—Aveiro Institute of Materials, Campus Universitário de Santiago, University of Aveiro, 3810-193 Aveiro, Portugal; morais.eduarda@ua.pt (E.S.M.); maragfreire@ua.pt (M.G.F.); cfreire@ua.pt (C.S.R.F.)

**Keywords:** furfural, deep eutectic solvents, metal halide salts, xylans, catalysis

## Abstract

The addition of alkali metal halide salts to acidic deep eutectic solvents is here reported as an effective way of boosting xylan conversion into furfural. These salts promote an increase in xylose dehydration due to the cation and anion interactions with the solvent being a promising alternative to the use of harsh operational conditions. Several alkali metal halides were used as additives in the DES composed of cholinium chloride and malic acid ([Ch]Cl:Mal) in a molar ratio of 1:3, with 5 wt.% of water. These mixtures were then used as both solvent and catalyst to produce furfural directly from xylan through microwave-assisted reactions. Preliminary assays were carried out at 150 and 130 °C to gauge the effect of the different salts in furfural yields. A Response Surface Methodology was then applied to optimize the operational conditions. After an optimization of the different operating conditions, a maximum furfural yield of 89.46 ± 0.33% was achieved using 8.19% of lithium bromide in [Ch]Cl:Mal, 1:3; 5 wt.% water, at 157.3 °C and 1.74 min of reaction time. The used deep eutectic solvent and salt were recovered and reused three times, with 79.7% yield in the third cycle, and the furfural and solvent integrity confirmed.

## 1. Introduction

The interest in alternative and renewable sources of chemicals, materials and energy has been growing in recent years. This trend is mostly due to the environmental impact associated with the overuse of petrochemical-derived products, notably CO_2_ emission and global warming [1], although instability in oil prices and supply might also be challenging in the long term. These arguments led to an increased interest in plant biomass to supply society with commodities previously attained from petroleum-based sources [2]. It is estimated that at least 30% of all chemicals should be derived from renewable resources by 2050 [3]. From the different chemicals that can be obtained from renewable resources, furfural is among the most relevant, with a world market of around 300,000 tons per year, while being identified by the US Department of Energy (DOE) as one of the top 12 value-added chemicals attained from biomass [4]. This compound is a precursor of other essential chemicals that are intermediates and/or solvents in industry, such as 2-methyltetrahydrofuran (2-Me-THF), furan, tetrahydrofuran (THF) and 2-methylfuran [5].

Furfural is produced from the dehydration of pentoses, and particularly from xylose dehydration in acidic media. Pentoses, in addition, are produced through the hydrolysis of hemicelluloses, which are amongst the most abundant components of lignocellulosic biomass (15–30 wt.%) [3,6]. Xylose is normally produced from one of the most common hemicelluloses, viz xylans [3,6]. Currently, furfural is mainly produced from agricultural wastes, such as corn cobs and sugar cane bagasse [6]. Most processes are carried out in aqueous media with homogeneous acidic catalysts, such as H_2_SO_4_ [7] and HCl [8], or heterogeneous catalysts, such as aluminium sulphate (Al_2_(SO_4_)_3_) [9] or tin tetrachloride (SnCl_4_) [10]. Numerous studies have focused on the production of furfural [11]; however, selectivity and solubility issues are bottlenecks that have limited the industrialization of the developed processes. These issues are mainly attributed to the nature of the catalysts. Acidic catalysts can cause the formation of side products such as humins, resulting from furfural condensation, while heterogeneous catalysts display low accessibility to biomass [5].

To diminish the rate and occurrence of side reactions, biphasic systems are frequently applied. Furfural is thus produced in aqueous media and continuously extracted to an organic phase to which it has a higher affinity [12]. Solvents such as methyl isobutyl ketone (MIBK) [13] and THF [14] have been used as the extracting phase. More recently, bio-based solvents have been used for the same purpose, such as, for example, γ-valerolactone (GVL) and 2-Me-THF [9,10,15,16,17]. Some solvents, such as GVL and dimethyl sulfoxide (DMSO), have been shown to suppress condensation reactions [18,19].

Ionic liquids (ILs) are alternative solvents often used to replace organic counterparts as reaction media. These neoteric solvents are low-temperature melting salts due to their ionic nature present negligible vapor pressure, outstanding chemical and thermal stability, and extended solvation ability [20]. ILs have been shown to display an important role in the synergistic transformation of biomass into chemicals [5]. In particular, ILs have shown promising results in the dehydration of furfural, either as reaction media [21] or as catalysts [22]. For example, Serrano-Ruiz et al. [22] designed ILs based on pyridinium/tetraethylammonium cations and functionalized them with butyl sulfonic acid groups and two different anions, namely tetrafluoroborate [BF_4_]^−^ and methyl sulfonate [MeSO_3_]^−^ [22]. These ILs were then used as catalysts for furfural production from xylose in different organic solvents achieving a maximum yield of 85% with THF at 80 °C for 1 h. Similarly, deep eutectic solvents (DES), composed of a hydrogen bond acceptor (HBA) and a hydrogen bond donor (HBD) [23], are a new class of alternative solvents that have been applied in the transformation of biomass into added-value chemicals [24]. DES are mixtures of two or more compounds that form an eutectic mixture strongly deviating from ideality, thus presenting a significant decrease in the melting temperature. These compounds also have a “designer solvent” character enabled by the proper selection of HBAs and HBDs [25,26]. They can be easily prepared by simply mixing, at appropriate ratio, the compounds required to create the desired eutectic mixture [24], and may display low toxicity if properly designed [27]. Recently, and due to the presented characteristics, some works have been published comprising the use of DES in furfural production [28,29,30,31]. DES composed of cholinium chloride ([Ch]Cl) and oxalic acid combined with Lewis acid catalysts, such as AlCl_3_·6H_2_O, has been applied combined with methyl isobutyl ketone (MIBK) in a biphasic system to convert xylans into furfural. This process resulted in 55.5% yield of furfural at 100 °C relative to the pristine xylans [28]. This same DES was applied in an aqueous solution (16.4 wt.% water) to transform palm oil fronds with ultra sounds (3 min), leading to a furfural yield of 56.5% at 120 °C for 60 min [29]. A biphasic system composed of an aqueous solution of [Ch]Cl:ethylene glycol with acetone allowed a furfural yield of 75% in the following conditions: 180 °C, 30 min, using 1.5 (*w*/*v*)% AlCl_3_ as a catalyst [30].

The topic of DES for furfural production has also been breached by our team [31]. In our work, microwave heating was applied with the DES composed of [Ch]Cl and malic acid, using xylan as substrate. A maximum furfural yield of 75.0% was achieved with only 2.5 min of reaction time by using the DES as both solvent and catalyst. In this work, bio-based solvents such as GVL and 2-Me-THF were used to boost yield, facilitate furfural production and to reuse the DES reaction media [31].

An interesting facet of the production of furfural is the role of alkali metal halides. It has been shown that the addition of these salts to solvents such as water and polar aprotic solvents can increase furfural yields [32,33]. Alkali metal halides have been used mostly with biphasic systems to increase the “salting-out” effect and thus increase furfural separation from the reaction media. Nonetheless, they also play an important role in the kinetics of xylose dehydration in acidic media [32,33]. Enslow et al. [32] found that the rate of xylose consumption is affected by both the nature of the cation and anion. Mellmer et al. [33] observed a similar trend in the increase in 5-hydroxymethylfurfural (HMF) yield when adding chloride ions to aprotic solvents such as GVL [33].

Considering that the use of alkali metal halides represents an effective way to increase furfural yields, in the present study, a selection of alkali metal halides were applied to the process we have previously optimized in deep eutectic solvents [31]. To the best of our knowledge the use of these alkali metal halides has not been studied with DES and could be an alternative to the use of harsher temperatures while decreasing the reaction times.

## 2. Results and Discussion

### 2.1. Metal Halide Selection and Screening

Considering that the use of alkali metal halides represents an effective way to increase furfural yields, in the present study, a selection of alkali metal halides, namely sodium chloride (NaCl), sodium bromide (NaBr), sodium iodide (NaI), potassium chloride (KCl), potassium bromide (KBr), potassium iodide (KI), lithium chloride (LiCl) and lithium bromide (LiBr), were applied to the process we have previously optimized [31], comprising the DES constituted by [Ch]Cl and malic acid, to address the possibility of further increasing the attained yields and without the need of using GVL as additive.

**Figure 1:**
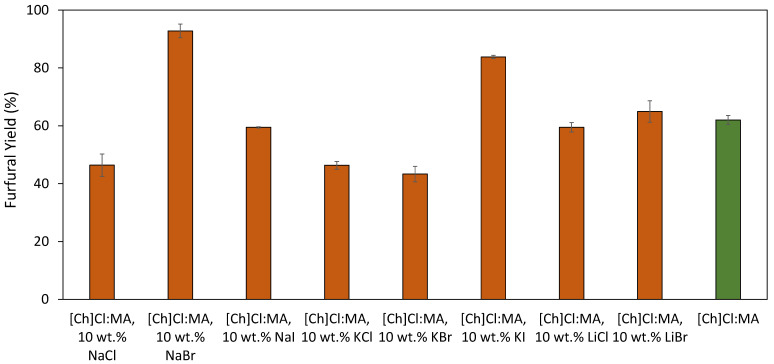
Screening of the different halide salts added at 10 wt.% to [Ch]Cl:MA (1:3), containing 5 wt.% H_2_O, in the furfural yield. The reactions occurred at 150 °C for 2.5 min with microwave heating. The last bar corresponds to the reference DES with no halide salt added.

**Figure 2:**
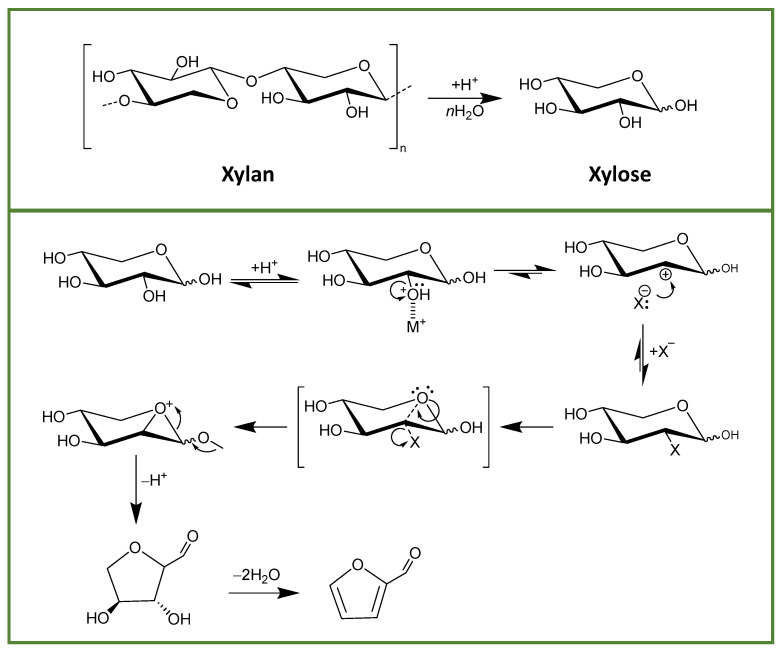
Scheme depicting the mechanism of furfural production in the presence of metal alkali halides. M^+^ represents the alkali metal cation and X^−^ represents the halide anion. Adapted from [32].

A screening was made using the different halide salts, added in a fixed concentration (10 wt.%) to the previously established best conditions for furfural production: [Ch]Cl:MA (1:3, molar ratio) with 5 wt.% of added water, at 150 °C for 2.5 min with microwave heating and solid–liquid (S/L ratio) of 0.05 [31]. The results obtained in these initial assays are presented in Figure 1. Furfural yields are affected, positively or negatively, with extreme values being observed with the addition of NaBr (up to 92.8% yield) and with the addition of NaCl (down to 43.3%) when compared with the reference conditions (no halide salt added, 62.0%). Overall, the addition of salts such as NaBr, NaI, KI and LiBr allow an increase in the furfural yield when compared with the reference conditions (no halide salt added). The mechanism for furfural production from xylan is initiated by the acidic hydrolysis of this compound into xylose. From there, the dehydration of xylose into furfural can occur in one of two ways: by ring opening to form the acyclic isomer of xylose followed by dehydration or by direct dehydration of either α- or β-xylopyranose. In this sense, Enslow et al. [32] have proposed an explanation for the role of metal halides in the dehydration taking place either at C2 (predominant) or C1, as depicted in Figure 2, for xylose dehydration in the C2 position. In the proposed mechanism, the metal cation is responsible for increasing the C–O–H and C–O–C bond lengths, through the interaction with the hydroxyl groups, and lowering the energy necessary to break such C–O bonds. Moreover, and as depicted in Figure 2, a reactive carbocation in C2 is formed after the loss of water that is stabilized by the formation of a halide. The use of metal cations with strong kosmotropic character will hinder the rehydration of this carbocation while the halide anions interact with electronegative regions of xylose, thus stabilizing the formed carbocation [32]. Nonetheless, the results reported here are not completely in line with the mentioned work [32] as in that case the xylose consumption was affected by the salt cation in the following order: no salt < K^+^ < Na^+^ < Li^+^.

In the present study, although NaBr outperforms KI, the salts containing Li^+^ do not enable higher furfural yields when compared with the maximum results attained for each cation. On the other hand, when comparing the anions studied, amongst the salts comprising Na^+^ and Li^+^, Br^–^ allows the best results; in the case of K^+^, the best results are attained in combination with I^−^. The latter result is in agreement with the work by Enslow and co-workers [32], which revealed increased xylose consumption in the following order: no salt < Cl^−^ < Br^–^ < I^–^. However, in that study [32], the assays were conducted with water as solvent. In these conditions, it was found that the halide salts were able to decrease water activity and therefore increase xylose dehydration rate. This was explained by the ability of the cations and anions to interact with water and thus reduce the water–water interactions [32]. In our system, water is present in low concentration (5.0 wt.%) and the effect of salt ions will mainly occur through the interaction with the DES components. The anions will mainly interact with the hydrogen-bond acceptor, that is, cholinium ion, whereas the cation will mainly interact with the chloride anion in the DES. These interactions then affect the xylose solvation and dehydration. Based on these overall trends it is clear that there is no general trend and that the effect of the salt ions is complex, with different trends appearing with different counterions.

**Figure 3:**
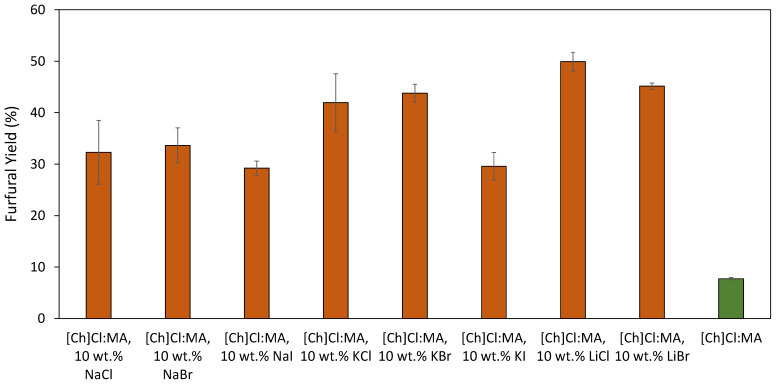
Screening of the different halide salts added at 10 wt.% to [Ch]Cl:MA (1:3), containing 5 wt.% H_2_O. The reactions occurred at 130 °C for 2.5 min with microwave heating. The last bar corresponds to the reference DES with no halide salt added.

It is important to note that in the assays using LiCl and LiBr we observed the formation of considerable amounts of insoluble side products. This could potentially mean that the reaction time was too long or the operating conditions too harsh, leading to the formation of side products in the cases in which xylose is dehydrated faster. Therefore, and to gage the behavior of the different salts in more detail, new assays were performed at a lower temperature of 130 °C, the results obtained being provided in Figure 3.

The results obtained reveal that at lower temperatures an alteration in the halide salts effect over furfural yields takes place. Furthermore, for most of the investigated DES, lower yields are obtained at lower temperatures, including a pronounced decrease seen with the reference DES. In these assays, an increase in furfural yield up to 49.9% was achieved with the addition of LiCl, while the lowest value corresponds to the DES reference (7.7%). These results reveal the catalytic effect of the different halide salts at lower temperatures, which is more noticeable when compared with the reference.

At lower temperatures, Li^+^ salts outperform those with both K^+^ and Na^+^, with K^+^ salts allowing a slightly higher yield when compared with salts comprising Na^+^, following the cation hydrogen-bond acidity. This trend is the opposite to what is observed in the literature [32]. As for the anion effect, at lower temperatures, I^−^ salts do not allow for higher furfural yields when compared to Cl^−^ and Br^−^ counterparts. Interestingly, the furfural yields attained with these two anions are very similar, being in agreement with the literature [32]. The positive effect of the addition of catalytic amounts of chloride salts for furans production was also observed in the work of Mellmer et al. [33] regarding the production of 5-hydroxymethylfurfural (5-HMF). It was revealed that the chloride anions stabilize the protonated transition states in the reactions, resulting in a tenfold increase in reactivity in aprotic solvents as GVL. The high concentration of the aprotic solvent is responsible for the formation of hydrophilic environments near the reactive hydroxyl group, stabilizing both the proton and chloride anions, and promoting fructose dehydration [33]. Overall, the attained results suggest that the halide salt cation effect is more susceptible to variations in different experimental parameters and solvents used, whereas the halide anions impact seems to be of a more universal nature with less synergetic effects taking place.

**Figure 4:**
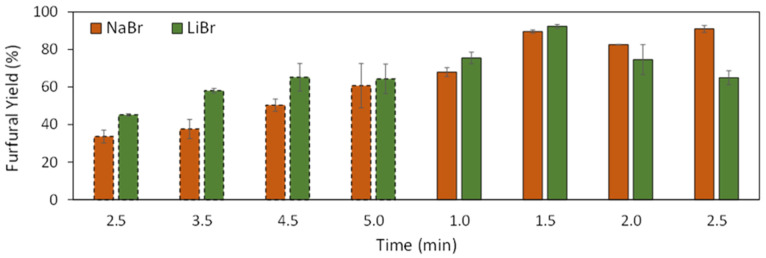
Furfural yield in solvents comprising [Ch]Cl:MA (1:3), 5 wt.% H_2_O and 10 wt.% of NaBr and LiBr at two different temperatures and different reaction times. Columns in dashed lines represent assays performed at 130 °C and columns in full line represent assays performed at 150 °C.

To further optimize furfural production yields, two of the best performing salts, namely LiBr and NaBr, were selected and applied at two different temperatures and variable reaction times under microwave heating. The respective results are displayed in Figure 4. The results obtained for these assays highlight the higher catalytic capability of LiBr at lower temperatures, with this salt outperforming NaBr in the assays carried out at 130 °C. Additionally, at higher temperatures, LiBr allows higher yields at lower reaction times. For reaction times of 2.0 and 2.5 min, the yields attained with LiBr decrease and the NaBr results surpass these values. This phenomenon may be due to the reaction’s kinetics, which are expected to be faster than LiBr is when compared with NaBr. This behavior is in line with the literature [32], in which the Li^+^ cation has a higher influence on the rate of xylose dehydration rate when compared with Na^+^ while also increasing the selectivity of the reaction towards furfural [32]. This increase in the reaction rate with the addition of LiBr results in a higher yield at lower reaction times; however, it also results in a decrease in yield as the reaction time goes on due to the degradation of furfural and the formation of side products. These side products are mostly humins resulting from furfural condensation [34]. The occurrence of these side reactions was already evidenced in our initial assays, i.e., by the appearance of precipitates. As for the assays in which NaBr was added, since the reaction is slower, the maximum yield is attained after a longer time.

### 2.2. Reaction Otimization through Response Surface Methodology

Due to the faster reaction rate and the possibility of working in a lower range of temperatures, the DES reaction media comprising LiBr was selected for further optimization in furfural production. Based on the different assays performed, temperature and time are key parameters in the optimization of the reaction for maximum furfural yield, in which furfural yields up to 90.9 and 92.3% can be obtained with DES reaction media comprising NaBr and LiBr, respectively (Figure 4).

**Figure 5:**
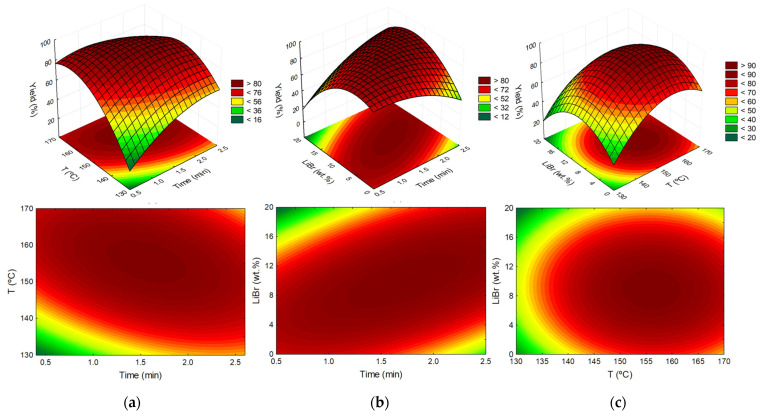
Response surface (top) and contour plots (bottom) of the furfural yield using [Ch]Cl:MA (1:3) + 5 wt.% H_2_O with the combined effects of: (**a**) temperature (°C) and time (min) with LiBr (wt.%) in the fixed condition of 10.0 wt.%; (**b**) LiBr content (wt.%) and time (min) with temperature (°C) in the fixed condition of 150°C; and (**c**) LiBr content (wt.%) and temperature (°C) with time (min) in the fixed condition of 1.5 min.

However, the amount of salt could also be a determining factor, being an additional parameter optimized in this work. In this sense, a Response Surface Method (RSM) was applied to optimize the parameters time (t, min), temperature (T, °C) and LiBr concentration (LiBr, wt.%). Concurrently, 2^3^ (3 factors and 2 levels) factorial planning was carried out [35], being described in detail in Tables S1 and S2. This methodology allows the optimization of the relationship between the response (furfural yield, %) and the independent variables/conditions. The obtained results were statistically analyzed with a confidence level of 95%. Student’s *t*-test was used to check the statistical significance of the adjusted data. The Statsoft Statistica 10.0^©^ software was considered for all statistical analyses and representing the response surfaces and contour plots. The influence of these three variables on furfural yield is illustrated in Figure 5, and in the Pareto chart given in Figure S1. Variance analysis (ANOVA) was applied to estimate the statistical significance of the variables and their interactions. The experimental points used in the factorial planning, the model equation, the furfural yield obtained experimentally and the respective calculated values, the correlation coefficients obtained, as well as all the statistical analyses are provided in Tables S2 and S3.

The results attained are in agreement with the values observed in the preliminary assays, revealing that the maximum furfural yield occurs between 1.2 and 2.2 min of reaction time, with temperatures ranging from 150 to 165 °C. Moreover, the optimal LiBr content is centered around 10.0 wt.%, which was the concentration used in the different preliminary assays. Nonetheless, it would be ideal to minimize the LiBr concentration while maintaining furfural yields to decrease the cost of the developed process. The pareto chart results (Figure S1) reveal that temperature and LiBr content are the most determining parameters in furfural yield. This behavior can be observed in Figure 5a,b, in which it is clear that good furfural yields are maintained through the length of the time axis. This phenomenon can be attributed to the efficiency of the microwave heating in the medium, which in turn narrows the reaction’s time window resulting in smaller differences observed between different time values. Nonetheless, a clear optimal region is defined for the relationship between the three variables.

According with the optimization performed, the optimal values determined for the three parameters are: 1.74 min of reaction time, 157.3 °C and 8.19 wt.% of LiBr. For this set of conditions, the furfural yield predicted is 92.2%. To address the veracity of the determined values, assays were conducted using the abovementioned conditions. The maximum furfural yield experimentally determined was 89.5 ± 0.3%, meaning that the obtained results confirm the adequacy of the statistical model. 

Overall, the maximum furfural yield obtained experimentally in the presence of LiBr under the optimized conditions (89.5 ± 0.3%), largely surpasses the yields achieved in our previous work [31] in which the same DES (with no halide salt added) was used. The results exceed even the performance of the DES in conjunction with GVL (75.0%) [31], while also having a faster reaction kinetic (1.74 vs. 2.5 min).

The yield values here achieved are higher than other previously established industrial processes, e.g., those utilized by companies such as Quaker Oats (<50%) and Dupont (61–84%) [36], while using milder conditions and a faster reaction time. These results highlight the potential of the developed process; however, a fair comparison with the industrial processes will only be possible after attempts of scaling up, technoeconomic analysis and life-cycle assessment studies. 

### 2.3. Furfural, Reaction Media and Catalyst Recovery and Reuse

The final step of this work focused on the recovery of pure furfural from the reaction media and the recovery and reuse of the reaction media (DES + LiBr) with the dissolved salt. The recovery of furfural was achieved through the direct distillation of this compound from the reaction media by reduced-pressure distillation in the following conditions: 50 ℃ and 6 mbar. With this strategy, 85.9% of furfural can be recovered with this simple and straightforward distillation. This value is in line with values attained in continuous processes [6] and is considerably higher when compared with established industrial processes [37]. Moreover, the purity of the attained furfural was accessed through ^1^H and ^13^C NMR (Figure S2). Both the NMR spectra and HPLC chromatogram for the recovered furfural do not present any extra peaks that could be attributed to the presence of secondary products. These results confirm that the obtained furfural is of high purity level.

**Figure 6:**
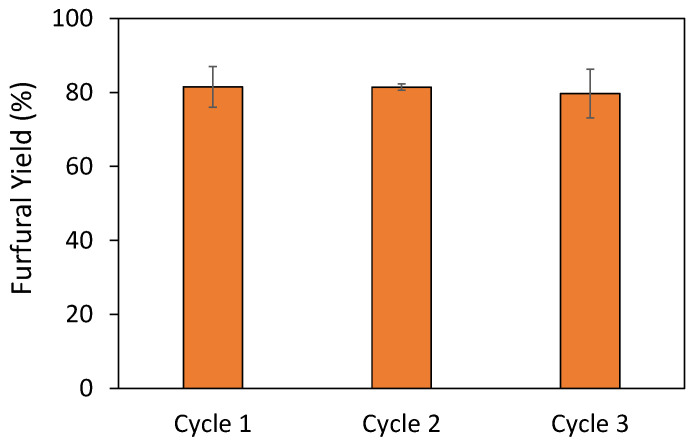
Furfural yields attained with different cycles of the DES [Ch]Cl:Malic Acid (1:3) + 5 wt.% H_2_O with 8.19% LiBr added at 157.3 ℃ and 1.74 min.

With this strategy it was possible to recycle the DES up to three times, as represented in Figure 6. In these assays a decrease in yield was observed in the first recycle (81.5 ± 5.5%); mostly due to the accumulation of insoluble side products, humins, in the reaction media, however, this yield is maintained throughout the next cycles (79.7 ± 6.6% in the third cycle). The integrity of DES attained in the last cycle was confirmed by comparison with freshly prepared DES. These results are given in Figure S4, showing that the recovered DES maintains its integrity. Thus, the possibility of reusing the DES + LiBr reaction media in the direct production of furfural from xylan clearly reduces the potential economic impact on the process of the use of LiBr.

## 3. Materials and Methods

### 3.1. Chemicals

Cholinium chloride ([Ch]Cl) (≥99% purity), furfural (≥99 % purity) and sodium iodide (NaI, 99% purtiy) were supplied by Sigma (Ronkonkoma, NY, USA). DL-Malic acid (MA, 99.5% purity) was obtained from Panreac (Barcelona, Spain) was obtained from Acros Organics (USA). Commercial beechwood xylan (9.39 wt.% humidity) was purchased from Apollo Scientific (Stockport, UK). Sodium chloride (NaCl, 99.5% purity) and sodium bromide (NaBr, 99% purity) were purchased from Fluka, Charlotte, NC, USA. Lithium Chloride (LiCl, 99% purity) gas attained from Merk and lithium bromide (LiBr, 99% purity) was attained from BDH Chemicals, Dubai, UAE. Potassium iodide (KI, 99.7 % purity) was purchased from Normapur (Radnor, PA, USA) and potassium chloride (KCl, 99.5% purity) was purchased from Chem Lab (Zedelgem, Belgium).

### 3.2. DES Preparation and Catalyst Addition

[Ch]Cl was used as the hydrogen bond acceptor (HBA) and malic acid (MA), as hydrogen bond donor (HBD). The water content of all the compounds was measured through a Metrohm 831 Karl Fisher coulometer. The DES was prepared by the weight measurement of the HBA and HBD at the desired molar ratio in sealed glass vials and the mixtures were then heated up to 70 °C and stirred for at least 1 h until a transparent liquid was formed. Mixtures were cooled down to room temperature and kept in sealed glass vials up until use. The water (5 wt.%) and the different salts were later added to the DES and stirred until complete homogenization/dissolution.

### 3.3. Xylan Hydrolysis and Dehydration to Furfural

Microwave-assisted reactions were utilized to produce furfural from xylans. Microwave irradiation was utilized since it is extremely advantageous when compared with conventional heating due to the extremely rapid heating of the reaction media. Moreover, it has been reported that it can increase the yields of carbohydrates dehydration reactions [38]. The microwave-assisted reactions were carried out in a Monowave 300 microwave synthesis reactor from Anton Paar (Graz, Austria). Heating was applied as fast as possible, with a heating time always below 1 min. The xylan/DES weight ratio was fixed at 0.05 in accordance with previous optimization [31]. The xylan and DES/salt mixtures were weighted and prepared in sealed vials (10 mL). Then, vials were placed in the microwave reactor at fixed stirring of 600 rpm. The reactions were carried out at 130 or 150 °C, and after the established reaction time (2.5 min) the samples were left to cool down, then diluted appropriately with deionized water and filtered through a nylon Whatman filter of 0.45 µm pore into appropriate HPLC glass vials. Triplicates were carried out in all assays.

### 3.4. Process Optimization through Response Surface Methodology

A RSM was applied to simultaneously analyze various factors (temperature, time and LiBr wt.%) and to identify the most significant parameters and their interaction, with the main goal of optimizing and promoting high furfural yields. A detailed description of the 2^3^ factorial planning used is provided in the Supplementary Materials Equations (S1) and (S2). The obtained results were statistically analyzed with a confidence level of 95%. Student’s *t*-test was used to check the statistical significance of the adjusted data. The adequacy of the model was determined by evaluating the lack of fit, the regression coefficient (and the F-value obtained from the analysis of variance (ANOVA) that was generated. The Statsoft Statistica 10.0^©^ software was used for all statistical analyses and representing the response surfaces and contour plots. From the interpretation of the RSM it was then possible to evaluate the best parameters for maximum furfural production. The attained conditions were then tested to validate the model.

### 3.5. Furfural Recovery and DES and Salt Recycling

Furfural was recovered from the DES media by direct distillation under reduced pressure. The distilled furfural was recovered, weighed and the furfural yield calculated. The recovered furfural was then redissolved in water, diluted and filtered through a nylon Whatman filter of 0.45 µm pore into HPLC glass vials. The recovered DES-based solvent was used in at least three more cycles of xylan conversion to verify the solvent recyclability on the furfural production yield.

The solvents stability was evaluated through ^1^H and ^13^C NMR. The obtained furfural was compared with a reference sample of furfural by ^1^H and ^13^C NMR. The ^1^H NMR and ^13^C NMR spectra were recorded using a Bruker Avance 300 at 300.13 MHz and 75.47 MHz, respectively, using deuterated water as solvent and trimethylsilyl propanoic acid (TMSP) as internal reference.

### 3.6. Furfural Quantification

The quantification of furfural in each sample was carried out by HPLC-DAD (Shimadzu, Kyoto, Japan, model PROMINENCE). HPLC analyses were performed with an analytical C_18_ reverse-phase column (250 mm length × 4.60 mm internal diameter), Kinetex 5 μm C_18_ 100 Å, from Phenomenex, Torrance, CA, USA. The mobile phase consisted of 20% of methanol and 80% of ultra-pure water. The separation was performed in isocratic mode, at a flow rate of 0.8 mL.min^−1^ and an injection volume of 10 μL. Furfural detection was carried out at 268 nm with a diode array detector (DAD). Each sample was analyzed at least in duplicate. The column oven and the autosampler were operated at a controlled temperature of 35 °C. Calibration curves were established with pure furfural dissolved in water. Furfural yield is expressed in moles of furfural in relation to the number of moles of xylose present in the xylan sample used.

## 4. Conclusions

In this work the addition of metal halide salts to the acidic-based DES [Ch]Cl:MA (1:3) + 5 wt.% H_2_O was investigated as a strategy to increase furfural yield directly from xylans. A screening of different salts was initially conducted in fixed conditions allowing the study and comparison of different anions (Cl^−^, Br^−^, I^−^) and cations (Na^+^, K^+^, Li^+^). From the conducted screening, LiBr allows the fastest and higher furfural yields, and was thus selected to be used in further optimization using a Response Surface Methodology approach.

The addition of halide salts is here shown to be an easy way of increasing furfural yield by boosting the performance of pre-existing systems. To the best of our knowledge, an 89.5 ± 0.3% furfural yield is the highest yield reported up to date in such a short time (1.74) and moderate temperature (157.3) of reaction.

The maximum furfural yield obtained experimentally in presence of LiBr under the optimized conditions was 89.5 ± 0.3%. The attained yield surpasses the yield attained in previous works using the same DES (75.0%) [31], with and without the addition of co-solvent γ-valerolactone, while also having a faster reaction kinetic (1.74 min vs. 2.5 min). Finally, the reaction media (DES + LiBr) can be reused at least three times (79.7% yield in the third cycle) without a major loss of yield. The obtained furfural is of high purity and the recovered DES maintains its integrity, further supporting the efficacy of the developed process.

The addition of halide salts is here shown to be an easy way of increasing furfural yields by boosting the performance of pre-existing systems. To the best of our knowledge, an 89.5 ± 0.3% furfural yield is the highest yield reported to date in such short reaction time. This is thus a promising system that offers several advantages when compared with other industrial processes, such as shorter reaction times and milder operating conditions. Nonetheless, the next steps for the development of a fully realized system that could compete with other established methodologies require the scale-up validation and its effect on the furfural yield, recovery and solvent reuse, as well as the need for carrying out technoeconomic analysis and life cycle assessment studies.

## Data Availability

Data is contained within the article or Supplementary Material.

## Supplementary Materials

The following are available online, Equation (S1): Response surface methodology polynomial equation; Equation (S2): Distance of the axial points to the central point; Table S1: 23 factorial planning; Table S2: Coded levels of independents variables used in the first and second factorial planning; Table S3: Regression coefficients of the predicted second-order polynomial model from factorial planning for the dependable variable of furfural yield; Figure S1: Pareto chart for the standardized main effects in the factorial planning for furfural yield optimization. Vertical line indicates the statistical significance of the effects; Figure S2: ^1^H and ^13^C NMR of the recovered furfural; Figure S3: ^1^H and ^13^C NMR the pure and recovered [Ch]Cl:Malic Acid (1:3).

## Sample Availability

Samples of the compounds xylans from beechwood are available from the authors.

## Abbreviations

DES: deep eutectic solvent; HBA: hydrogen bond acceptor; HBD: hydrogen bond donor; RSM: response surface methodology; IL: ionic liquid; [Ch]Cl: cholinium chloride; MA: Malic Acid; MW: microwave.

## References

[1] Machado G., Leon S., Santos F., Lourega R., Dullius J., Mollmann M.E., Eichler P. (2016). Literature Review on Furfural Production from Lignocellulosic Biomass. Nat. Resour..

[2] Sheldon R.A. (2016). The E factor 25 years on: The rise of green chemistry and sustainability. Green Chem..

[3] Fiorentino G., Ripa M., Parthenope N. (2016). Chemicals from biomass: Technological versus environmental. Biofuels Bioprod. Biorefining.

[4] Werpy T., Petersen G., Aden A., Bozel J., Holladay J., White J., Manheim A. (2004). Top Value Added Chemicals from Biomass Volume I—Results of Screening for Potential Candidates from Sugars and Synthesis Gas Top Value Added Chemicals.

[5] Chen B., Peng Z., Li C., Feng Y., Sun Y., Tang X. (2021). Catalytic Conversion of Biomass to Furanic Derivatives with Deep Eutectic Solvents. ChemSusChem.

[6] Li X., Jia P., Wang T. (2016). Furfural: A Promising Platform Compound for Sustainable Production of C4 and C5 Chemicals. ACS Catal..

[7] Rong C., Ding X., Zhu Y., Li Y., Wang L., Qu Y., Ma X., Wang Z. (2012). Production of furfural from xylose at atmospheric pressure by dilute sulfuric acid and inorganic salts. Carbohydr. Res..

[8] Yemiş O., Mazza G. (2011). Acid-catalyzed conversion of xylose, xylan and straw into furfural by microwave-assisted reaction. Bioresour. Technol..

[9] Yang T., Zhou Y.H., Zhu S.Z., Pan H., Huang Y.B. (2017). Insight into Aluminum Sulfate-Catalyzed Xylan Conversion into Furfural in a Γ-Valerolactone/Water Biphasic Solvent under Microwave Conditions. ChemSusChem.

[10] Wang W., Ren J., Li H., Deng A., Sun R. (2015). Direct transformation of xylan-type hemicelluloses to furfural via SnCl4 catalysts in aqueous and biphasic systems. Bioresour. Technol..

[11] Zhao Y., Lu K., Xu H., Zhu L., Wang S. (2021). A critical review of recent advances in the production of furfural and 5-hydroxymethylfurfural from lignocellulosic biomass through homogeneous catalytic hydrothermal conversion. Renew. Sustain. Energy Rev..

[12] Luo Y., Li Z., Li X., Liu X., Fan J., Clark J.H., Hu C. (2019). The production of furfural directly from hemicellulose in lignocellulosic biomass: A review. Catal. Today.

[13] Sweygers N., Harrer J., Dewil R., Appels L. (2018). A microwave-assisted process for the in-situ production of 5-hydroxymethylfurfural and furfural from lignocellulosic polysaccharides in a biphasic reaction system. J. Clean. Prod..

[14] Yang Y., Hu C.W., Abu-Omar M.M. (2012). Synthesis of furfural from xylose, xylan, and biomass using AlCl 3·6H2O in biphasic media via xylose isomerization to xylulose. ChemSusChem.

[15] Li W., Zhu Y., Lu Y., Liu Q., Guan S., Chang H.M., Jameel H., Ma L. (2017). Enhanced furfural production from raw corn stover employing a novel heterogeneous acid catalyst. Bioresour. Technol..

[16] Lin Q., Li H., Wang X., Jian L., Ren J., Liu C., Sun R. (2017). SO42−/Sn-MMT Solid Acid Catalyst for Xylose and Xylan Conversion into Furfural in the Biphasic System. Catalysts.

[17] Cai C.M., Nagane N., Kumar R., Wyman C.E. (2014). Coupling metal halides with a co-solvent to produce furfural and 5-HMF at high yields directly from lignocellulosic biomass as an integrated biofuels strategy. Green Chem..

[18] Lu H.Z., Bai J.F., Yan F., Zhang X.Y., Jin Y., Wang J.Y., Chen P., Zhou M.D. (2021). Oxidation of 5-hydroxylmethylfurfural to 2, 5-furandicarboxylic acid catalyzed by magnetic MnO2-Fe3O4 composite oxides. Ranliao Huaxue Xuebao/J. Fuel Chem. Technol..

[19] Zhang Z., Huber G.W. (2018). Catalytic oxidation of carbohydrates into organic acids and furan chemicals. Chem. Soc. Rev..

[20] Ventura S.P.M., E Silva F.A., Quental M.V., Mondal D., Freire M.G., Coutinho J.A.P. (2017). Ionic-Liquid-Mediated Extraction and Separation Processes for Bioactive Compounds: Past, Present, and Future Trends. Chem. Rev..

[21] Zhang L., Yu H., Wang P. (2013). Solid acids as catalysts for the conversion of d-xylose, xylan and lignocellulosics into furfural in ionic liquid. Bioresour. Technol..

[22] Serrano-Ruiz J.C., Campelo J.M., Francavilla M., Romero A.A., Luque R., Menéndez-Vázquez C., García A.B., García-Suárez E.J. (2012). Efficient microwave-assisted production of furfural from C 5 sugars in aqueous media catalysed by Brönsted acidic ionic liquids. Catal. Sci. Technol..

[23] Abbott A.P., Capper G., Davies D.L., Rasheed R.K., Tambyrajah V. (2003). Novel solvent properties of choline chloride/urea mixtures. Chem. Commun.

[24] Khandelwal S., Tailor Y.K., Kumar M. (2016). Deep eutectic solvents (DESs) as eco-friendly and sustainable solvent/catalyst systems in organic transformations. J. Mol. Liq..

[25] Tang B., Ho K. (2013). Recent developments in deep eutectic solvents in chemical sciences. Mon. Chem.

[26] Zhang Q., De Oliveira Vigier K., Royer S., Jérôme F. (2012). Deep eutectic solvents: Syntheses, properties and applications. Chem. Soc. Rev..

[27] Dai Y., van Spronsen J., Witkamp G.-J., Verpoorte R., Choi Y.H. (2013). Natural deep eutectic solvents as new potential media for green technology. Anal. Chim. Acta.

[28] Zhang L.-X., Yu H.H., Yu H.H., Chen Z., Yang L. (2014). Conversion of xylose and xylan into furfural in biorenewable choline chloride–oxalic acid deep eutectic solvent with the addition of metal chloride. Chin. Chem. Lett..

[29] Basil C., Loong T., Yeong T., Kui C., Fong L., Mei I., Chew L. (2021). Nonsevere furfural production using ultrasonicated oil palm fronds and aqueous choline chloride-oxalic acid. Ind. Crop. Prod..

[30] Chen Z., Wan C. (2019). A novel deep eutectic solvent/acetone biphasic system for high-yield furfural production. Bioresour. Technol. Rep..

[31] Morais E.S., Freire M.G., Freire C.S.R., Coutinho J.A.P., Silvestre A.J.D. (2020). Enhanced conversion of xylan into furfural using acidic deep eutectic solvents with dual solvent and catalyst behavior. ChemSusChem.

[32] Enslow K.R., Bell A.T. (2015). The role of metal halides in enhancing the dehydration of xylose to furfural. ChemCatChem.

[33] Mellmer M.A., Sanpitakseree C., Demir B., Ma K., Elliott W.A., Bai P., Johnson R.L., Walker T.W., Shanks B.H., Rioux R.M. (2019). Effects of chloride ions in acid-catalyzed biomass dehydration reactions in polar aprotic solvents. Nat. Commun..

[34] Delbecq F., Wang Y., Muralidhara A., El Ouardi K., Marlair G., Len C. (2018). Hydrolysis of Hemicellulose and Derivatives—A Review of Recent Advances in the Production of Furfural. Front. Chem..

[35] Khuri A.I., Mukhopadhyay S. (2010). Response surface methodology. Wiley Interdiscip. Rev. Comput. Stat..

[36] Lee C.B.T.L., Wu T.Y. (2021). A review on solvent systems for furfural production from lignocellulosic biomass. Renew. Sustain. Energy Rev..

[37] Xing R., Qi W., Huber G.W. (2011). Production of furfural and carboxylic acids from waste aqueous hemicellulose solutions from the pulp and paper and cellulosic ethanol industries. Energy Environ. Sci..

[38] Guenic S.L., Delbecq F., Ceballos C., Len C. (2015). Microwave-assisted dehydration of D-xylose into furfural by diluted inexpensive inorganic salts solution in a biphasic system. J. Mol. Catal. A Chem..

