# Supplementary material for: Enhanced Furfural Production in Deep Eutectic Solvents Comprising Alkali Metal Halides as Additives
Source: Molecules. 2021 Dec 4;26(23):7374. doi: 10.3390/molecules26237374 (PMC8659074; doi:10.3390/molecules26237374)
Supplement: Supplementary file 1 [file molecules-26-07374-s001.zip › molecules-1450498-supplementary.pdf]

Supplementary Material

Enhanced Furfural Production in Deep Eutectic Solvents Comprising Alkali Metal Halides as Additives

Eduarda S. Morais, Mara G. Freire, Carmen S. R. Freire and Armando J. D. Silvestre *

Chemistry Department, CICECO—Aveiro Institute of Materials, Campus Universitário de Santiago, University of Aveiro, 3810-193 Aveiro, Portugal
* Correspondence: armsil@ua.pt

Process optimization – response surface methodology (RSM)

In a 2^k surface response methodology there are k factors that contribute to a different response, and the data are treated according to a second order polynomial equation according to equation S1:

$$y = \beta_0 + \sum \beta_i X_i + \sum \beta_{ii} X_i^2 + \sum_{i<j} \beta_{ij} X_i X_j \qquad \text{(S1)}$$

where y is the response variable and β_0, β_i , β_{ii} and β_{ij} are the adjusted coefficients for the intercept, linear, quadratic and interaction terms, respectively, and X_i and X_j are independent variables. This model allows the drawing of surface response curves and through their analysis the optimal conditions can be determined [35]. The 2^3 factorial planning has been defined by the central point (zero level), the factorial points (1 and −1, level one) and the axial points (level α) The axial points are encoded at a distance α from the central point, according to equation S2:

$$\alpha = (2^k)^{1/4} \qquad \text{(S2)}$$

Table S1. 2^3 factorial planning.

Experiment	χ_1	χ_2	χ_3
**1**	-1	-1	-1
**2**	1	-1	-1
**3**	-1	1	-1
**4**	1	1	-1
**5**	-1	-1	1
**6**	1	-1	1
**7**	-1	1	1
**8**	1	1	1
**9**	-1.68	0	0
**10**	1.68	0	0
**11**	0	-1.68	0
**12**	0	1.68	0
**13**	0	0	-1.68
**14**	0	0	1.68
**15**	0	0	0
**16**	0	0	0
**17**	0	0	0
**18**	0	0	0
**19**	0	0	0
**20**	0	0	0

Table S2. Coded levels of independents variables used in the first and second factorial planning.

Studied parameters	Symbol	Level				
		Axial	Factorial	Central	Factorial	Axial
		-1.68	-1	0	1	1.68
**Time (min)**	Time	0.7	1.0	1.5	2.0	2.3
**Temperature (°C)**	T	133.20	140.00	150.00	160.00	166.80
**LiBr (wt.%)**	LiBr	1.600	5.000	10.000	15.000	18.400

Table S3. Regression coefficients of the predicted second-order polynomial model from factorial planning for the dependable variable of furfural yield.

	Regression coefficients	Standard deviation	t-student (10)	P-value
**Interception**	-1591.5559	623.2909	-2.5534	< 0.05
**Time**	122.4043	111.0500	1.1022	0.2961
**Time ^{2}**	-11.5116	10.4901	-1.0973	0.2981
**Temperature**	20.2637	7.9735	2.5413	< 0.05
**Temperature ^{2}**	-0.0613	0.0262	-2.3376	< 0.05
**LiBr**	2.6500	10.9676	0.2416	0.8139
**LiBr ^{2}**	-0.2733	0.10490	-2.6061	< 0.05
**Time × Temperature**	-0.7052	0.7028	-1.0033	0.3393
**Time × LiBr**	2.3337	1.4057	1.6601	0.1278
**LiBr × Temperature**	-0.0082	0.0704	-0.1168	0.9092

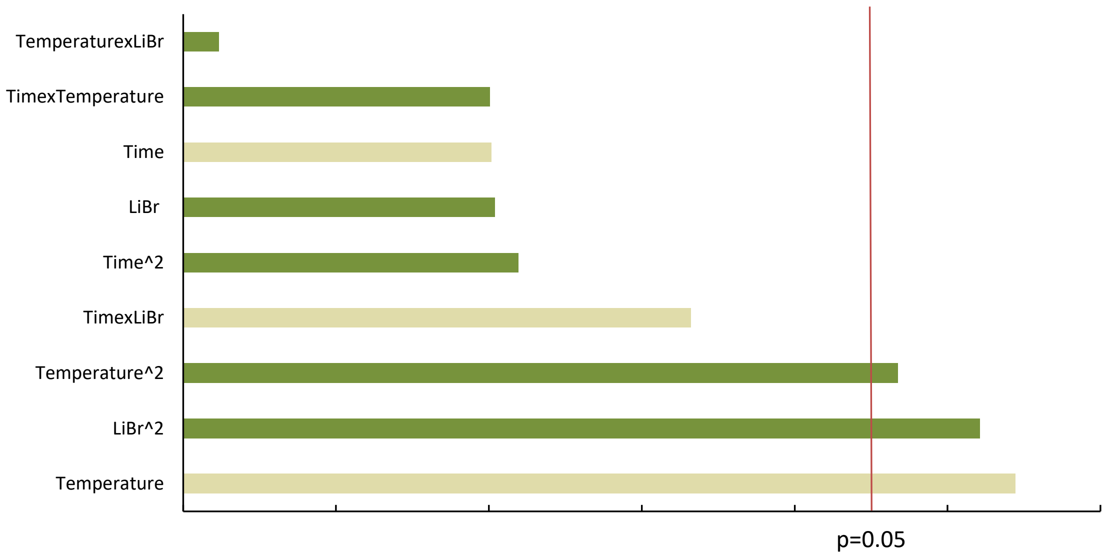

Figure S1. Pareto chart for the standardized main effects (positive ( ) and negative ( )) in the factorial planning for furfural yield optimization. Vertical line indicates the statistical significance of the effects.

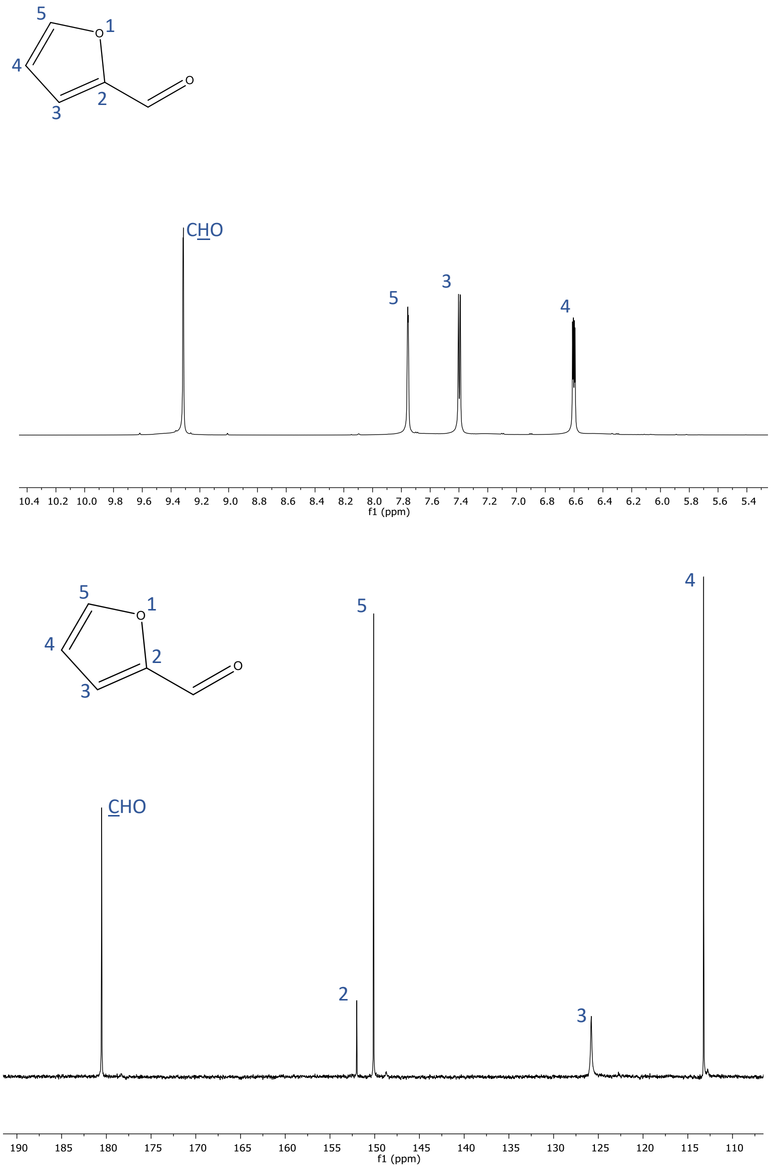

Figure S2. ^{1}H and ^{13}C NMR of the recovered furfural.

^{1}H NMR (D_2O, 300 MHz, [ppm]): δ 9.30 (d, 1H, 2-C<u>H</u>=O), 7.73 (dd, 1H, H-5), 7.41 (dd, 1H, H-3), 6.64 (dd, 1H, H-4). ^{13}C NMR (D_2O, 75.47 MHz, [ppm]): δ 180.50 (2-<u>C</u>H=O), 152.05 (C-2), 150.18 (C-5), 125.80 (C-3), 113.20 (C-4).

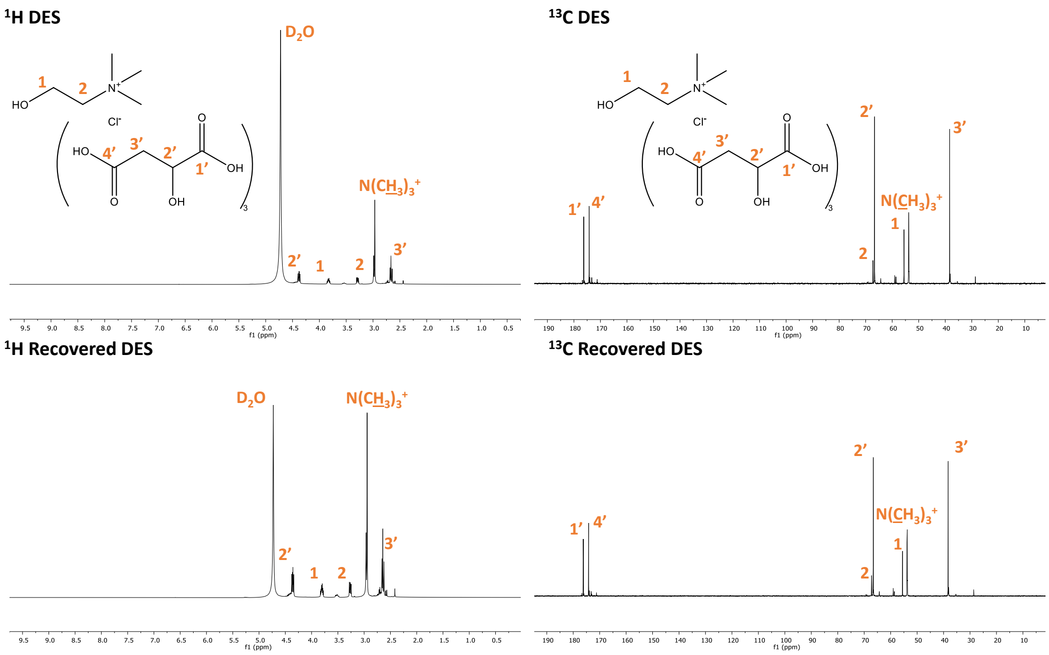

Figure S3. ^{1}H and ^{13}C NMR the pure and recovered [Ch]Cl:Malic Acid (1:3).
